# Conserved and species-specific oxylipin pathways in the wound-activated chemical defense of the noninvasive red alga *Gracilaria chilensis* and the invasive *Gracilaria vermiculophylla*

**DOI:** 10.3762/bjoc.8.30

**Published:** 2012-02-21

**Authors:** Martin Rempt, Florian Weinberger, Katharina Grosser, Georg Pohnert

**Affiliations:** 1 Institute for Inorganic and Analytical Chemistry, Lessingstr. 8, Friedrich-Schiller-University, D-07743 Jena, Germany, Tel: +493641948171; Fax +493641948172; 2 Leibniz-Institut für Meereswissenschaften, IFM-GEOMAR, Düsternbrooker Weg 20, D-24105 Kiel, Germany

**Keywords:** activated chemical defense, invasive species, oxylipins, prostaglandins, red algae, regulation

## Abstract

Chemical defense of the invasive red alga *Gracilaria vermiculophylla* has been studied and compared to that of the noninvasive but related *Gracilaria chilensis*. Both species rely on a wound-activated chemical defense that makes them less attractive to the herbivorous sea snail *Echinolittorina peruviana*. The chemical stress response of both species was monitored by LC–ESIMS-based metabolic profiling and revealed commonalities and differences. Both algae rely on a rapid lipoxygenase mediated transformation of arachidonic acid to known and novel oxylipins. Common products are 7,8-dihydroxyeicosatetraenoic acid and a novel eicosanoid with an unusual γ-lactone moiety. Several prostaglandins were predominantly formed by the invasive species*.* The role of some of these metabolites was investigated by surveying the attachment of *E. peruviana* on artificial food containing the respective oxylipins. Both algae species are defended against this general herbivore by 7,8-dihydroxyeicosatetraenoic acid, whereas the prostaglandins and the novel oxylipins were inactive at naturally occurring concentrations. The role of different oxylipins in the invasive potential of *Gracilaria spp.* is discussed.

## Introduction

The red alga *Gracilaria chilensis* is native along the Chilean coast and is commercially farmed for the production of agar hydrocolloids [1]. Since the alga can be easily planted and harvested, this crop is exploited by the local population widely along the Chilean coast. However, no uncontrolled invasion has been reported to date. In contrast, the related *Gracilaria vermiculophylla*, which is native to the Northwest Pacific, is an invasive species in the north Atlantic and represents a potential threat to the native flora and fauna [2]. The species is spreading in habitats that are dominated by generalist herbivores and suffers only minor losses due to grazing. It has been hypothesized that introduced species are most likely to be successful in the presence of generalist herbivores if they are well defended. Here we address the chemical defenses of the two related algae, which are both particularly rich in oxylipins that are predominantly produced after physical wounding [3–4]. For *G. chilensis*, the role of specific oxylipins in the chemical defense against epiphytism has been demonstrated. This alga has two major lines of defense, including a rapid wound-activated production of oxylipins and a slower induced defense involving the up regulation of phospholipases and lipoxygenases and subsequent fatty-acid transformation [3,5]. Among the up-regulated metabolites, arachidonic acid derived hydroxylated and dihydroxylated fatty acids are most prominent, with 7,8-dihydroxyeicosatetraenoic acid (7,8-di-HETE (**3**)) being the most active metabolite in the chemical defense against epiphytism. Recent work indicates that the invasive *G. vermiculophylla* also relies on wound-activated transformations of arachidonic acid for its chemical defense. Bioassays with the generalist isopod grazer *Idotea baltica,* which is found in the areas in which *G. vermiculophylla* is invasive, revealed that among all the detected oxylipins a minor prostaglandin is responsible for the chemical defense [6]. Both *Gracilaria* form common (hydroxylated and dihydroxylated fatty acids **3** and **4**) and unique (prostaglandins **1** and **2** in *G. vermiculophylla*) arachidonic acid derived oxylipins. Using a novel chemometric evaluation of metabolic profiles, we followed arachidonic acid metabolism in both species and identified known and novel oxylipins [6]. We asked ourselves whether the species-specific oxylipin profiles may explain a different chemical defense and may thus be a prospective cause for the invasive potential of *G. vermiculophylla*. We selected the sea snail *Echinolittorina peruviana* as a model herbivore to monitor the role of the oxylipins in the chemical defence of the algae. This snail is highly abundant on Chilean and Peruvian coasts where *G. chilensis* is native and can reach population densities of up to 2000 individuals/m^2^ [7]. *E. peruviana* is known to graze on biofilms as well as on macroalgae and can thus be employed in the monitoring of ecologically relevant interactions.

## Results and Discussion

**Figure 1:**
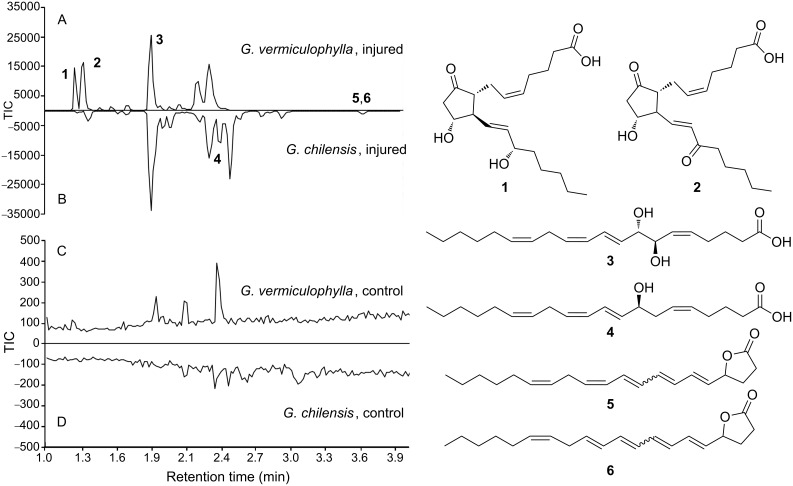
UPLC–ESIMS-based metabolic profiles of *Gracilaria vermiculophylla* (A) and *Gracilaria chilensis* (B) induced by grinding and incubation for 5 min at rt. In comparison, control runs with intact algal material that was boiled to prevent enzyme activity before extraction are given in (C) and (D). Total-ion-count (TIC) traces (ESI-negative mode) are shown; note: TIC normalized to 35000 counts in the wounded and to 500 counts in the chromatographic profiles of the control. The structures of the major metabolites are given, and the retention times of **5** and **6** are indicated; these metabolites do not show up in the ESI negative mode.

As reported previously, wounding of *G. chilensis* triggers the pronounced formation of arachidonic acid derived oxylipins, including the most dominant 7,8-di-HETE (**3**) and 8-hydroxy-eicosatetraenoic acid (8-HETE (**4**)) [3]. This was verified by comparative LC–ESIMS and LC–ESIMS/MS analysis of algae that were wounded by grinding in a mortar, incubated at room temperature for five minutes, and extracted (wounded), versus algae that were boiled before grinding and extraction (Figure 1). Boiling prevents enzymatic reactions during work-up and results in the extraction of metabolites found in the intact algae (hereafter termed “intact”). Only trace levels of prostaglandins were detected in the wounded algae. The boiling of samples leads to a suppression of enzyme activity that would occur during wounding and thus allows profiling of the secondary metabolites in the intact cells. Independent experiments showed that the oxylipins in question are stable and survive the brief boiling without detectable degradation. Previously we were able to verify that the feeding activity of herbivores triggers metabolic responses similar to the introduced wounding procedure in *G. vermiculophylla*, thereby justifying this treatment as appropriate for the monitoring of wound-activated defences [6].

**Scheme 1:**
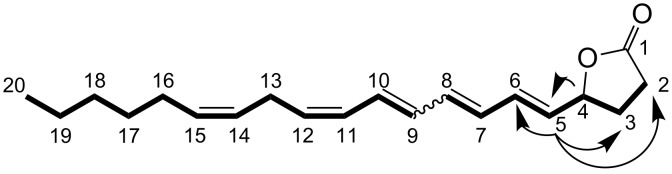
5-((1*E*,3,5*E*,7*Z*,10*Z*)-hexadeca-1,3,5,7,10-pentaenyl)dihydrofuran-2(3*H*)-one (**5**) with ^1^H–^1^H COSY (bold bonds) and relevant HMBC (arrows) correlations.

By using a precursor-directed search for novel arachidonic acid derived oxylipins [6], we detected an additional signal of an arachidonic acid derived metabolite with a characteristic UV spectrum of a conjugated tetraene. Purification yielded about 2.3 mg of an unstable metabolite, which was submitted to MS, 1D and 2D-NMR analysis. The molecular formula C_20_H_28_O_2_ was determined by HRMS–ESI ([M + Na]^+^ calcd for 323.1986; found, 323.1982). ^1^H–^1^H COSY allowed us to follow the entire spin system of the metabolite, including 10 olefinic protons, 7 CH_2_, 1 CH_3_ and 1 CH groups (Scheme 1, spectra are shown in Supporting Information File 1).

**Scheme 2:**
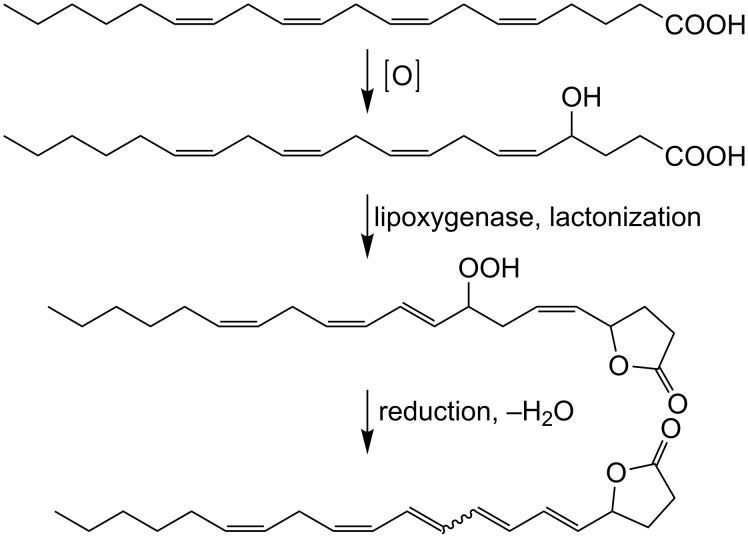
Suggested pathway for the biosynthesis of **5**.

In combination with HMBC data all hydrogen and carbon signals were assigned, but the stereochemistry of the double bond C7/C8 remained open. The signal at 5.0 ppm in the proton range (proton at C4) and the resonance at 80.45 ppm (^13^C, HMBC, C4) as well as HMBC and ^1^H–^1^H COSY signals led to the assignment of a γ-lactone ring (Scheme 1). HMBC correlations from the carbon C4 to H2, H3, H5 and H6 clarified the location of the lactone. Two protons on an isolated *Z*-configured double bond were observed at 5.44–5.31 ppm. The stereochemistry of the remaining conjugated tetraene system was assigned by analysis of the coupling constants according to [8]. The hydrogen at position-5 couples to H6 with a coupling constant of 15.6 Hz, which is indicative of an *E*-double bond. The H9 to H10 coupling constant of 14.6 Hz indicates an *E*-configuration as well, while the H11 to H12 coupling of 11.45 Hz indicated a *Z*-configuration. The double-bond configuration between H7 and H8 could not be resolved due to the substantial overlap of the signals. An isomer of **5** bearing also a conjugated tetraene unit was isolated and tentatively assigned to a stereoisomer with an *E*-configured double bond between C11 and C12 and open stereochemistry of the C7/C8 bond. Despite the stereocenter at C4, **5** and **6** were optically inactive and exhibited no signal in circular dichroism measurements. Both isomers **5** and **6** were already detected in the crude extracts, but also after work-up and during storage in CDCl_3_ further isomerization was observed. The biosynthetic origin of **5** and **6** from arachidonic acid was proven by applying the stable isotope-labeled precursor [^2^H_2_]-arachidonic acid [6] to the frozen powder before incubation and extraction. Analysis of the resulting mass spectra showed incorporation rates of 56% and confirmed an efficient transformation to **5** and **6**. The racemic nature of **5** and **6** indicates the involvement of a nonenzymatic step during their formation. A possible biosynthetic pathway to **5** and **6** (Scheme 2) could involve an oxidation at C4. This oxidation could proceed in a similar way to that required for the introduction of the 7-OH group in 7,8-di-HETE (**3**). A lipoxygenase-mediated transformation of arachidonic acid to an 8-hydroperoxide, followed by reduction and elimination of water along with isomerization of the double bonds, could then provide the substrate for lactonization. Since **5** and **6** were configurationally unstable during purification, subsequent isomerizations may lead to the observed products. Other mechanisms, involving the formation of an extended conjugated double-bond system and the attack of the carboxylic acid on an intermediate double bond at C4 can, however, not be excluded.

Wounding of *G. vermiculophylla* also led to pronounced changes in the metabolic profile (Figure 1, [6]). As in *G. chilensis* the major up-regulated metabolite was 7,8-di-HETE (**3**), which was detected in very similar concentrations in both species. 8-HETE (**4**) was only present in trace amounts. In contrast to *G. chilensis*, the wound-activated production of the prostaglandins **1** and **2** is a lot more pronounced. It can be estimated that these metabolites can account for more than 50% of the total oxylipins, if integrals of the corresponding signals from the oxylipins are evaluated while neglecting possibly differences in the response factors in ESIMS. The novel lactones **5** and **6** were also detected in this species.

Quantification of the oxylipins bears some uncertainty, since the absolute and relative amounts detected after wounding vary strongly. This can be attributed to a certain phenotypic plasticity, but since multiple samples from the same individual also exhibited fluctuations, it is more likely that the wounding procedure triggers a highly uncontrolled action of lipases, lipoxygenases, oxidases [3], and cyclooxygenases [9], which results in the observed varying amounts of oxylipins. In all experiments the quantities of prostaglandins were high in *G. vermiculophylla*, while only traces were detected in *G. chilensis* (Figure 1). To determine the amount of potential defense metabolites to be used in bioassays, the mean concentrations of the metabolites were determined in triplicates from batches that were also used for bioassays. The mean values of the determined amounts of prostaglandins from *G. vermiculophylla* were used for the bioassays. Since the amounts of 7,8-di-HETE (**3**), **5**, and **6** were similar in *G. vermiculophylla* and *G. chilensis*, the mean concentrations of these metabolites in both species were employed. We thus tested 15-keto-PGE_2_ **2** as a 2 µg/g treatment in agar, PGE_2_ **1** (2.6 µg/g agar), 7,8-di-HETE **3** (1.7 µg/g agar), and a 1/1 mixture of **5** and **6** (total 8.2 µg/g agar).

**Figure 2:**
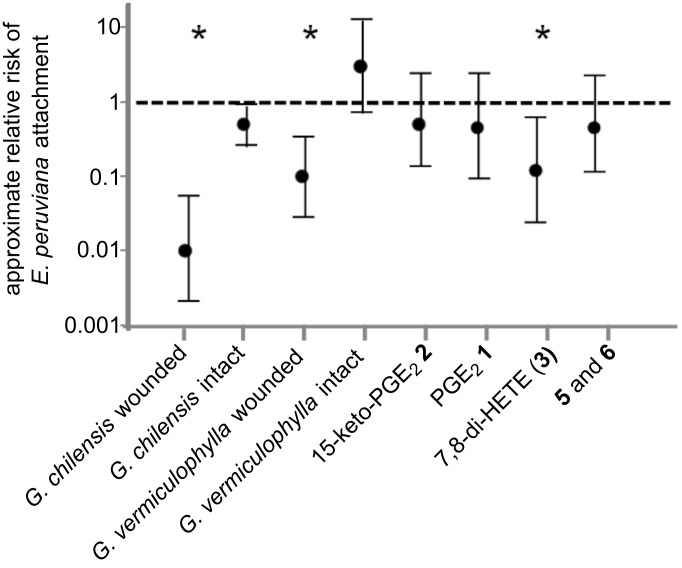
Relative risk (mean ± 95% confidence interval) of *Echinolittorina peruviana* attachment on surfaces treated with *Gracilaria chilensis* and *Gracilaria vermiculophylla* extracts obtained before and after wounding. In addition, the relative risk of attachment to pure compounds in natural concentrations is given. Asterisks indicate significant effects when examined with the χ²-test, as described in Fisher and van Belle [10].

To evaluate the response of the sea snail *E. peruviana* towards the algal compounds, we developed a new bioassay based on the avoidance reaction of the snail. When the snails were put on a Petri dish that was partially filled with agar prepared with seawater, the snails attached to this layer of jellified seawater. When the plate was turned upside down after seven minutes, the snails stayed attached to the surface (control). In contrast, when the agar contained extracts or active chemicals, the snails retracted and fell down directly after the Petri dish was turned. After counting the attached and fallen individuals, we could determine the risk of attachment from the odds ratio {(snails on extract, attached)/(snails on extract, unattached)}/{(snails on solvent control, attached)/(snails on solvent control, unattached)}, as described in [10]. A risk of 1 would indicate that the same amount of snails remained on the agar in the treatment compared to the solvent control; values smaller than 1 indicate that snails avoided the surface with the treatment, which in nature would also result in cessation of feeding. When the agar was treated with methanolic extracts of intact *G. chilensis* and *G. vermiculophylla* no reduced attachment was observed. In contrast, when extracts from wounded algae of both species were incorporated, a significantly reduced proportion of snails remained attached to the agar compared to the control (Figure 2). In these experiments we selected the concentration of the embedded algal extract to be equivalent to that in the fresh (wet weight) algae. The intensity of this response was clearly dose-dependent. For example, the extract of wounded *G. chilensis* caused reduced snail retraction at a 0.1-fold dilution, and only an insignificant reduction was observed when the extract was applied in a 0.01-fold natural concentration (data not shown). Both species seem thus to be defended against the generalist herbivore, exploiting a strategy based on the formation of active metabolites after wounding.

To identify the compounds responsible for the observed activated defense, we undertook a large-scale (2 kg) extraction of *G. vermiculophylla* and purified the up-regulated metabolites using silica chromatography and preparative reversed-phase HPLC. The purified compounds (**1**, **2**, **3** and a ca. 1:1 mixture of **5** and **6**) were incorporated into agar matrices in natural concentrations and the response of the snails was tested by using the attachment bioassay, as described above. Of the tested metabolites only 7,8-di-HETE (**3**) was active when applied at concentrations corresponding to those detected in wounded *G. vermiculophylla*. The isolated prostaglandins and the new lactones (**5** and **6**) did not exhibit any activity at natural concentrations (Figure 1) or 10-fold higher amounts (data not shown). Interestingly, 7,8-di-HETE (**3**), a compound that also proved to be the most-active metabolite in the chemical defense of *G. chilensis* against epiphytes [3], can explain the entire activity of the *G. vermiculophylla* and *G. chilensis* crude extracts against *E. peruviana*. This metabolite thus protects the invasive and the native species from herbivory by the snails. Apparently the higher diversity of oxylipins from *G. vermiculophylla* cannot explain its success as an invasive species if only grazing by the generalist *E. peruviana* is concerned. Nevertheless, prostaglandins play important roles in the chemical defense against the generalist herbivorous isopod *Idotea baltica* [6]. While *E. peruviana* is a herbivore that co-occurs with *G. chilensis*, *I. baltica* is a generalist that is often encountered in regions in which *G. vermiculophylla* spreads in an invasive manner. Thus our observation would explain that the algae are defended against herbivores that are co-occurring, by a conserved defense mechanism (this study), while the invasive potential of *G. vermiculophylla* can be explained by prostaglandins that are only detected in this species [6].

## Conclusion

The chemical defense of *Gracilaria spp*. against *E. peruviana* is stimulated by tissue disruption and proceeds via the release of arachidonic acid derived oxylipins. We demonstrate that the structurally diverse oxylipins detected in *Gracilaria spp*. play individual roles in the chemical defense against different herbivores. The dihydroxylated fatty acid 7,8-di-HETE is generally active against co-evolved herbivores, while prostaglandins support an invasive success of the algae.

## Experimental

**General.** NMR spectra were recorded on Bruker Avance 400 MHz or 600 MHz spectrometers, and chemical shifts are given in ppm relative to the chemical shift of the solvent signal of CDCl_3_. HRMS were recorded on a MAT 95 XL (Thermo Finnigan, Bremen, Germany) equipped with an ESI probe. LC–MS measurements were performed on an UPLC–MS system equipped with a 2996 PDA detector and a Q-tof micro ESIMS (Waters, Manchester, UK). For separation of the analytes, a BEH C18 column (2.1 × 50 mm, particle size 1.7 µm) was used. LC on a semipreparative column was carried out by using a LC-8A liquid chromatography system from Shimadzu (Duisburg, Germany) equipped with a SPD-10AV UV–vis detector. A Licro Chart® 250-10 Purosphere® RP-18 endcapped column (particle size 5µm) supplied by Merck (Darmstadt, Germany) was used for separation. HPLC separation on an analytical scale was performed on the same LC system equipped with a Synergi MAX-RP 250 × 4.6 mm column (particle size 4 µm) from Phenomenex (Macclesfield, UK). Optical rotations were measured on a Jasco (Groß-Umstadt, Germany) 1030 polarimeter at 589 nm. Circular-dichroism spectra were recorded on a Jasco P810 instrument. Analytical thin-layer chromatography was carried out by using silica gel 60 F_254_ plates. Compounds were detected by ceric ammonium molybdate (CAM) staining. Solvents and reagents were purchased from Sigma Aldrich (Deisenhofen, Germany).

**Biological material.** Laboratory strains of *Gracilaria vermiculophylla* (originating from El Jedidah, Morocco, available from the culture collection of algae and protozoa, Oban, Scotland) and *Gracilaria chilensis* (strain CR14, originating from Caldera, Chile) were cultivated as described in Weinberger et al. [5]. The strains were used in order to generate extracts for the quantification of oxylipins, UPLC profiling, and bioassays. For large-scale purification *G. vermiculophylla* was collected at Norderhafen, Germany. The snail *Echinolittorina peruviana* was collected at Las Cruces, Chile, transferred to the lab and maintained in seawater at 10 °C on a diet of *Ulva sp*.

**Extraction and bioassays.** Herbivory was mechanically simulated by grinding of algae (10 g) in a mortar and subsequent incubation for 5 min at rt before extraction with MeOH (2 mL). After filtration through cellulose, the extracts were mixed in a glass Petri dish with hot (60 °C) seawater (10 mL) containing 1.5% agar (Sigma Aldrich, Deisenhofen, Germany) and immediately cooled to 10 °C, resulting in a layer of jellified seawater agar that contained the extract at natural concentrations, assuming a 100% extraction success; in reality these concentrations will be slightly below the natural concentrations. For the investigation of intact algae, algae (10 g) were boiled for 1 min in seawater to denature the enzymes. The algae were subsequently shock frozen in liquid nitrogen and treated as described above. Independent treatments showed that the oxylipins, besides **5** and **6**, are stable under these conditions (data not shown). Solvent controls were prepared in the same way with pure MeOH. To test for deterrence, 20 individuals of *E. peruviana* were placed upon agar containing either algal extracts or solvent only. After 7 min the Petri dishes with agar and snails were turned upside down, and the individuals that remained attached under these conditions were counted. The approximate relative risk of snail attachment to the agar-containing extract was calculated as described above. To test specific metabolites, pure compounds (15-keto-PGE_2_ **2**: 2 µg/g agar; PGE_2_ **1**: 2.6 µg/g algae; 7,8-di-HETE (**3**): 1.7 µg/g algae; **5** and **6** as 1/1 mixture: 8.2 µg/g agar) were dissolved in acetonitrile and incorporated into the agar as described above for the extracts. Solvent controls were run with corresponding concentrations of acetonitrile.

**Metabolic profiling.** Methanolic extracts for metabolic profiling were prepared as described above and investigated with UPLC–MS. UPLC solvent A (water-acidified with 0.1% formic acid (v/v) and 1% acetonitrile (v/v)); solvent B (acetonitrile). Gradient: 0 min at 0% B; 0.5 min 50% B; 5 min 100% B; 5.5 min 100% B; 6 min 0% B. Total ion counts are shown in Figure 1.

**Large-scale extraction.** About 2 kg of *G. vermiculophylla* (wet weight) with minor impurities were harvested at Nordhafen, Germany. The biomass was frozen in liquid nitrogen and crushed in the cold state to a fine powder by using a household blender. The biomass was allowed to come to rt before 5 L of ethylacetate were added. Extraction was performed for 10 h under permanent shaking in brown glass bottles at 10 °C. Subsequently, the solvent was filtered and a second 5 h extraction with 5 L of ethylacetate was carried out. The pooled organic extracts were dried over Na_2_SO_4_ and the solvent was removed under reduced pressure. The residue was then immediately prepurified by flash column chromatography on silica gel (solvent system: chloroform/isopropanol/acetone 80:20:15 (v/v/v)). The oxylipin-rich fraction (verified by UPLC–MS) was then evaporated and portions of the residue (300 mg) were separated by semipreparative HPLC (solvent A: water acidified with 0.1% formic acid (v/v) and solvent B: methanol. Gradient: 0 min at 25% B; 18 min 60% B; 22 min to 80% B; 24 min to 100% B; 29 min at 100% B; 32 min 25% B). Further purification of metabolites **5** and **6** was performed by analytical HPLC (solvent A: water acidified with 0.1% formic acid (v/v) and 1% acetonitrile (v/v); solvent B: acetonitrile. Gradient: 0 min at 65% B; 11 min 100% B; 14 min at 100% B; 14.1 min to 65% B; 17 min 65% B).

**Incorporation of labeled arachidonic acid.** 2-[^2^H_2_]-arachidonic acid [6] (2 mg) was suspended in water (15 µL) in a 1.5 mL Eppendorf cap by using a laboratory vortexer. Frozen, powdered algae (5 mg; see above) were added. The mixture was vortexed and allowed to warm to rt. The mixture was maintained at rt for 5 min and subsequently MeOH/water (300 µL; 2:1 v/v) was added. After vortexing and centrifugation, the supernatant was directly used for UPLC–MS analysis. As a control, the same procedure was followed in the absence of the labeled fatty acid.

**Quantitative analysis.** The extracted or commercially available compounds were used to create three calibration standards for each analyte, covering the concentration range detected in the extract from wounded algae. A calibration curve was recorded on the LC–MS system by repeated (3 ×) injection of each standard. Analyte peaks were displayed by their pseudo-molecular-ion trace, and the peak areas were used for quantification.

**Structure elucidation of known metabolites.** The identification of compounds **1**–**4** was achieved by comparison of the obtained NMR and MS spectra by co-injection with authentic standards [3]. Stereochemistry of PGE_2_ was verified with CD spectroscopy in comparison with an authentic standard.

**Spectroscopic data of 5.** 5-((1*E*,3*E*,5*E*,7*Z*,10*Z*)-hexadeca-1,3,5,7,10-pentaenyl)dihydrofuran-2(3*H*)-one (**5**) was obtained as light-yellow oil. The molecular formula C_20_H_28_O_2_ was determined by HRMS–ESI, [M + Na]^+^ calcd for 323.1986; found, 323.1982. The UV–vis absorption maxima at 293 nm, 306 nm and 321 nm can be explained by a conjugated tetraene-structure. ^1^H NMR (600 MHz, CDCl_3_) δ (the numbering of atoms refers to Scheme 1) 0.89 (t, *J* = 6.8 Hz, 3H, H20), 1.40–1.24 (m, 6H, H17, H18, H19), 2.10–1.97 (m, 3H, H3, H16), 2.45–2.33 (m, 1H, H3), 2.60–2.51 (m, 2H, H2), 2.95 (dd, *J* = 6.9, 7.1 Hz, 2H, H13), 5.00 (dt, *J* = 7.1, 15.0 Hz, 1H, H4), 5.37 (dtd, *J* = 12.4, 6.9, 1.8 Hz, 1H, H14), 5.46 (dtd, *J* = 12.6, 7.3, 1.4 Hz, 1H, H15), 5.46 (dt, *J* = 7.8, 10.5 Hz, 1H, H12), 5.68 (dd, *J* = 6.62, 15.64 Hz, 1H, H5), 6.08–6.03 (dd, *J* = 11.1, 14.7 Hz, 1H, H11), 6.26–6.16 (m, 2H, H7, H9), 6.36–6.33 (m, 2H, H6, H8), 6.57 (m, 1H, H10); ^13^C NMR (150 MHz, CDCl_3_) δ 176.75 (C1), 135.25 (C6), 133.21 (C8), 132.24 (C9), 131.84 (C12), 131.01 (C7), 130.73 (C15), 129.60 (C10), 129.41 (C5), 128.50 (C11), 126.87 (C14), 80.45 (C4), 31.51 (C18), 29.27 (C19), 28.87 (C3), 28.54 (C2), 27.25 (C16), 26.31 (C13), 22.55 (C17), 14.04 (C20).

## Supporting Information

File 1 Spectra for metabolites **5** and **6**.
