# Supplementary material for: Conserved and species-specific oxylipin pathways in the wound-activated chemical defense of the noninvasive red alga Gracilaria chilensis and the invasive Gracilaria vermiculophylla
Source: Beilstein J Org Chem. 2012 Feb 21;8:283–9. doi: 10.3762/bjoc.8.30 (PMC3302091; doi:10.3762/bjoc.8.30)
Supplement: File 1 — Spectra for metabolites 5 and 6. [file Beilstein_J_Org_Chem-08-283-s001.pdf]

Supporting information

for

Conserved and species-specific oxylipin pathways in the wound-activated chemical defense of the noninvasive red alga *Gracilaria chilensis* and the invasive *Gracilaria vermiculophylla*

Martin Rempt[1], Florian Weinberger[2], Katharina Grosser[1], Georg Pohnert*[1]

Address: [1]Institute for Inorganic and Analytical Chemistry, Lessingstr. 8, Friedrich-Schiller-University, D-07743 Jena, Germany, Tel: +493641948171; Fax +493641948172 and [2]Leibniz-Institut für Meereswissenschaften, IFM-GEOMAR, Düsternbrooker Weg 20, D-24105 Kiel, Germany

Email: Georg Pohnert - Georg.Pohnert@uni-jena.de

*Corresponding author

Spectra for metabolites 5 and 6.

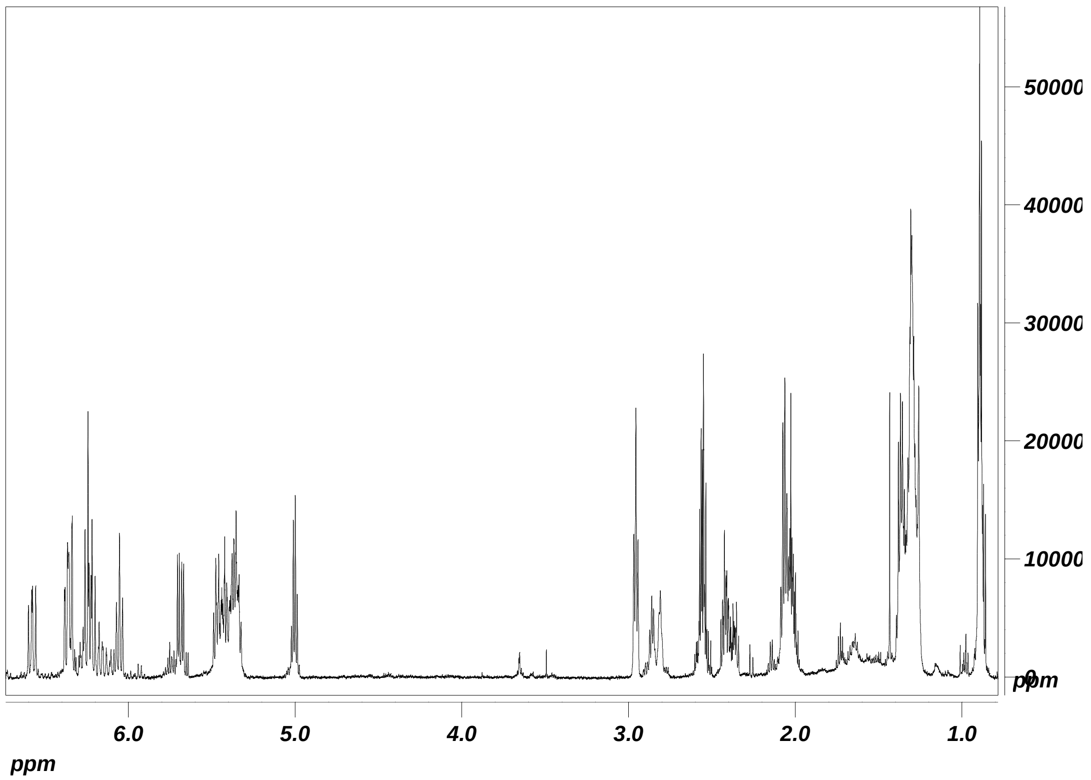

Figure S1: ^{1}H NMR spectrum of compound **5**.

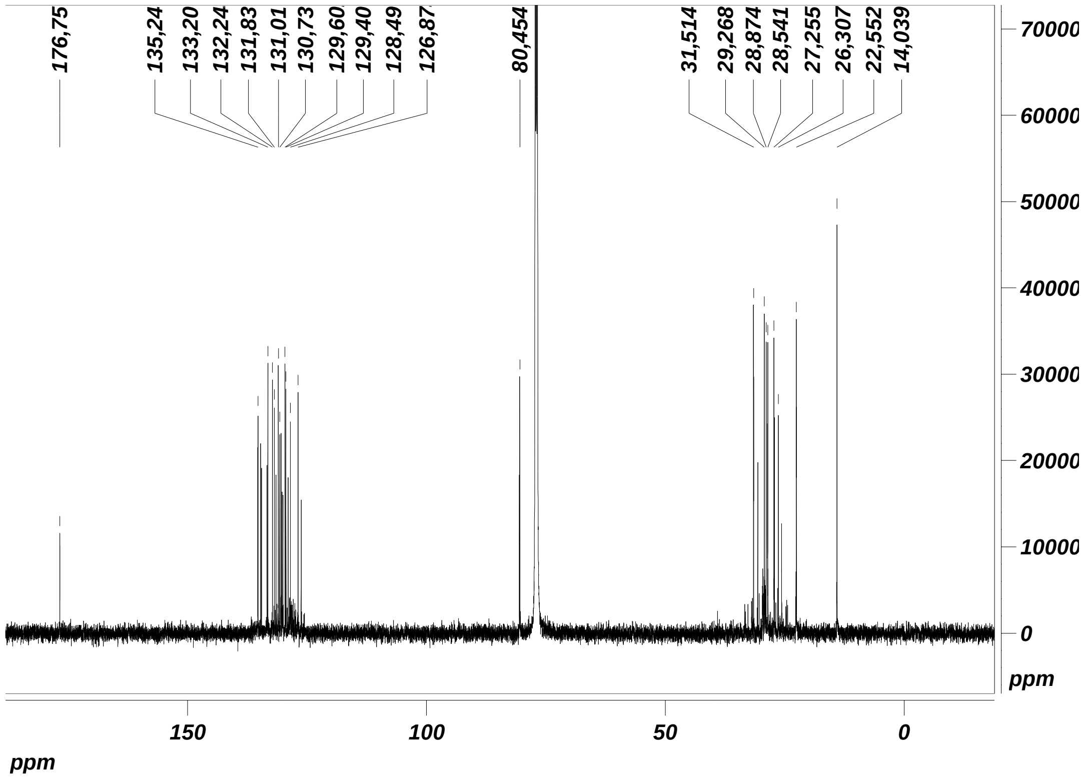

Figure S2: ^{13}C NMR spectrum of compound **5**.

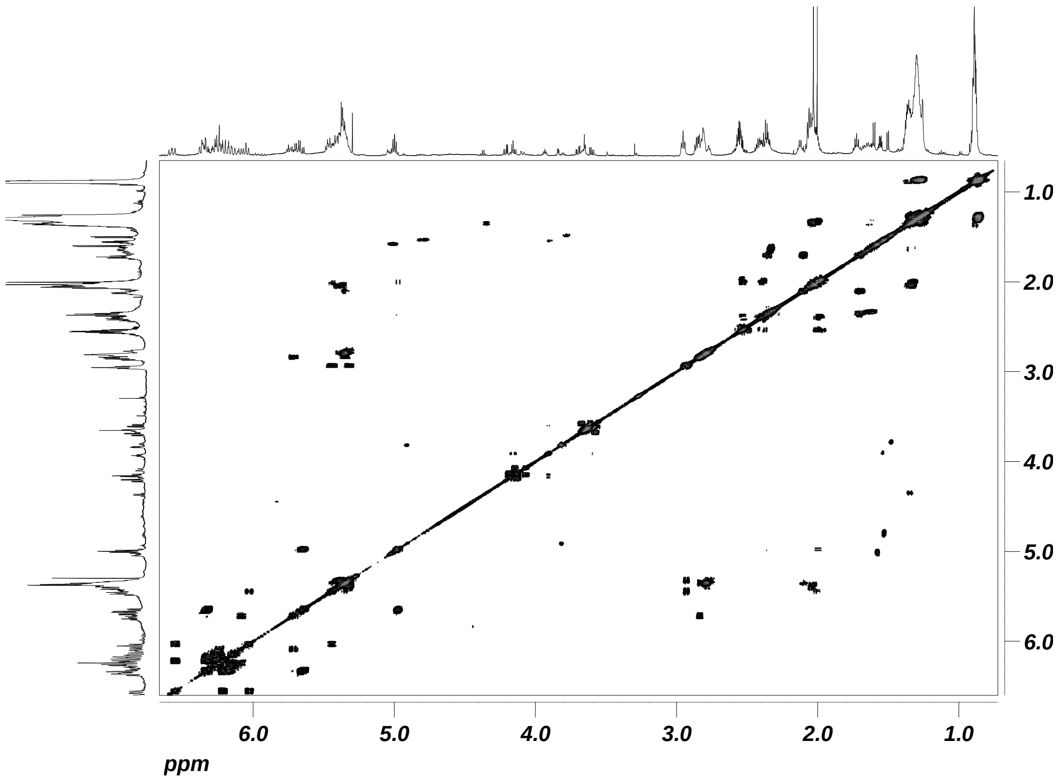

Figure S3: $^{1}H/^{1}H$-COSY spectrum of compound **5**.

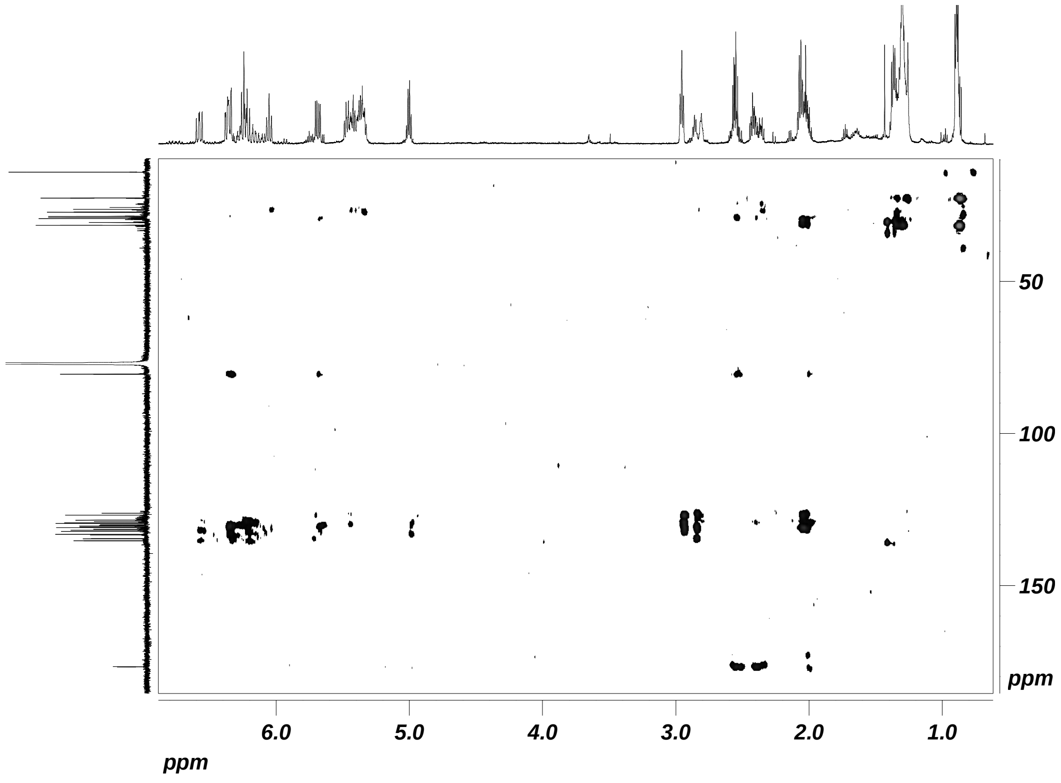

Figure S4: HMBC spectrum of compound **5**.

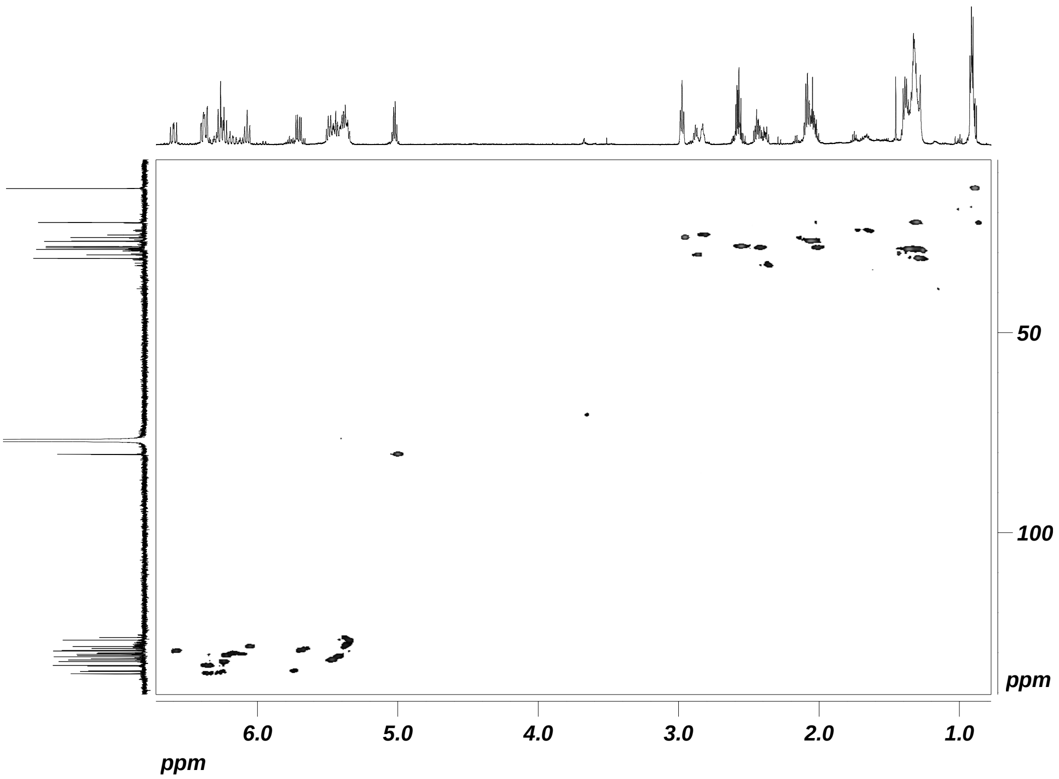

Figure S5: HSQC spectrum of compound **5**.

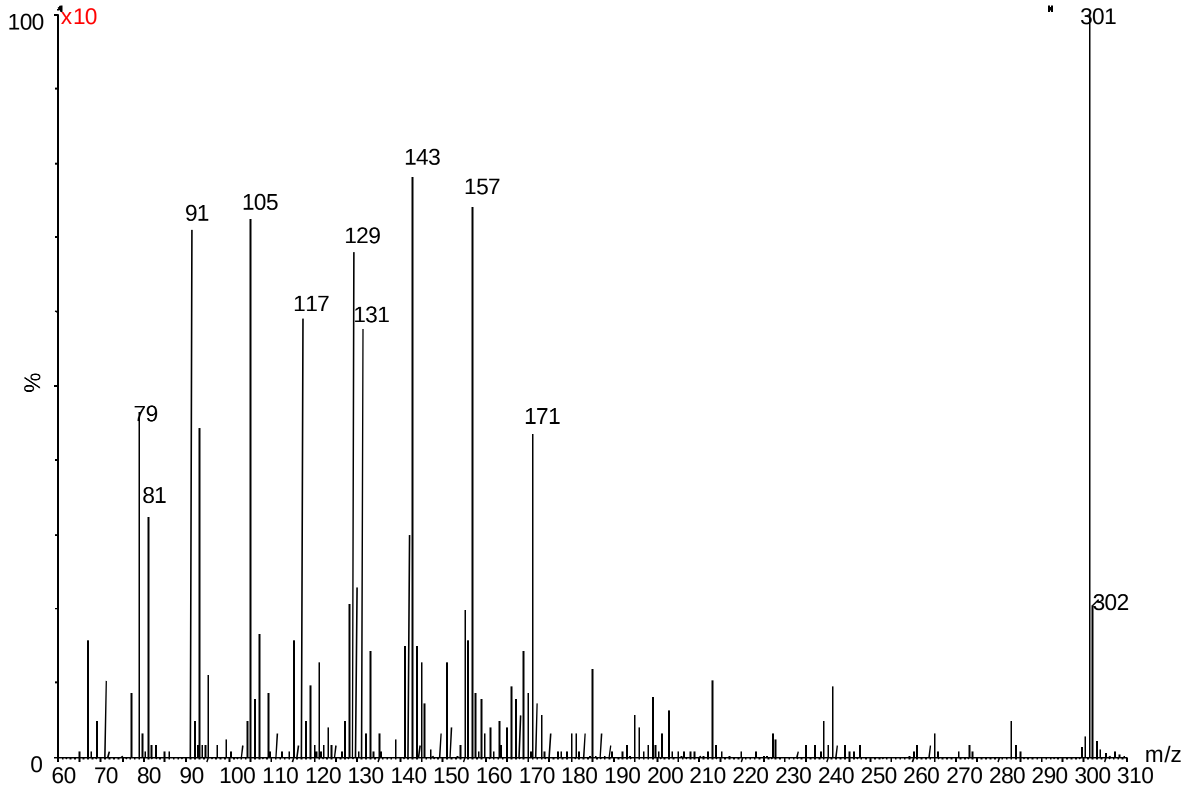

Figure S6: ESI-MS/MS spectrum of compound **5** (target mass 301 amu).

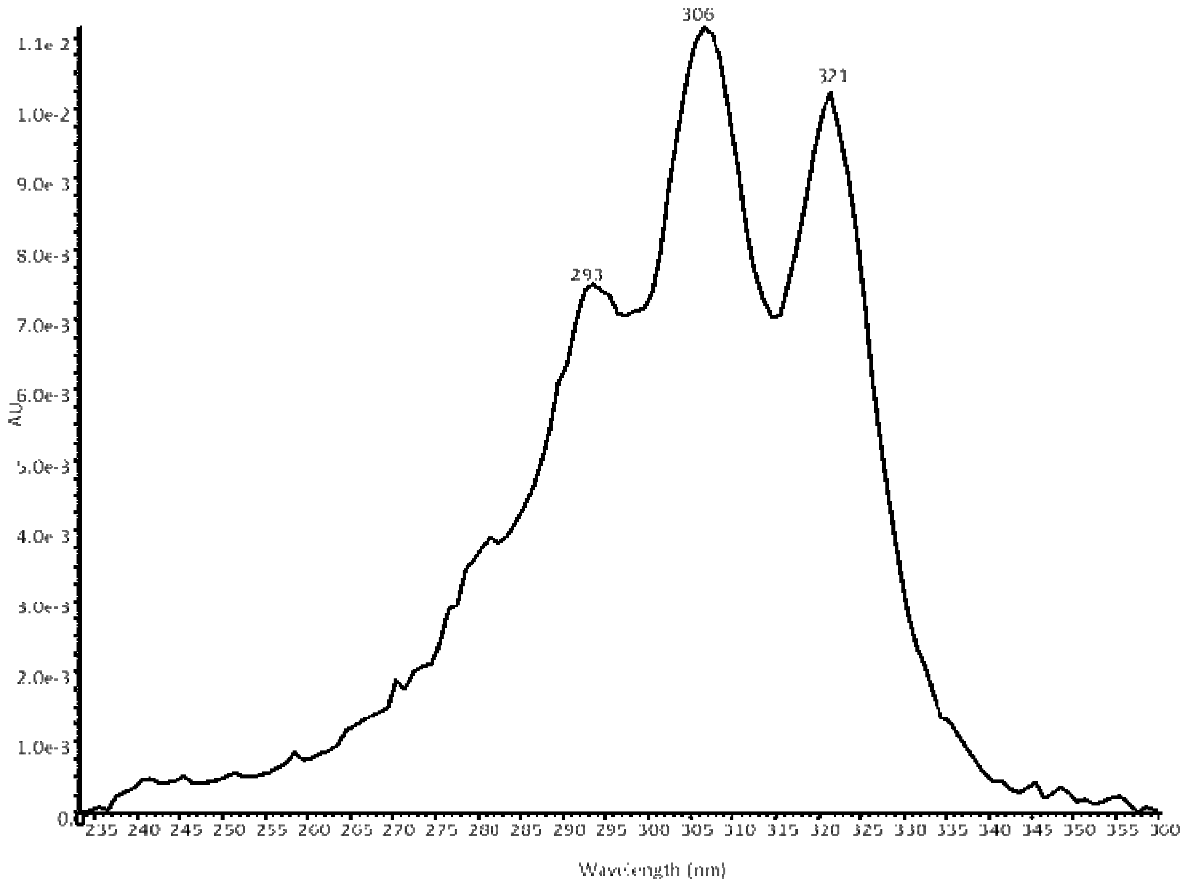

Figure S7: UV–vis spectrum of compound **5** in acetonitrile.

5-((1*E*,3*E*,5*E*,7*E*,10*Z*)-hexadeca-1,3,5,7,10-pentaenyl)dihydrofuran-2(3*H*)-one (**6**) was obtained as a light-yellow oil. The analysis of the *J*-couplings between H11 and H12 revealed the *trans* character of the double bond in comparison to compound **5**. Furthermore, a lambda (max) shift of about 2 nm in the UV–vis confirmed this assignment.

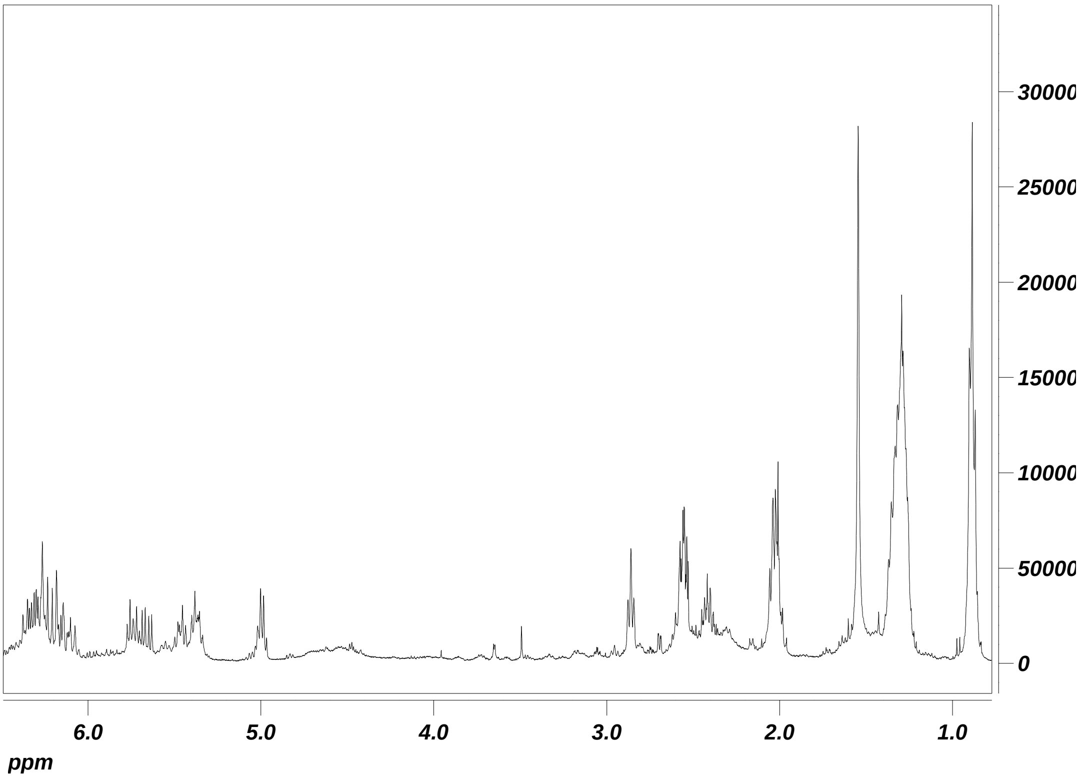

Figure S8: ^{1}H NMR spectrum of compound **6**.

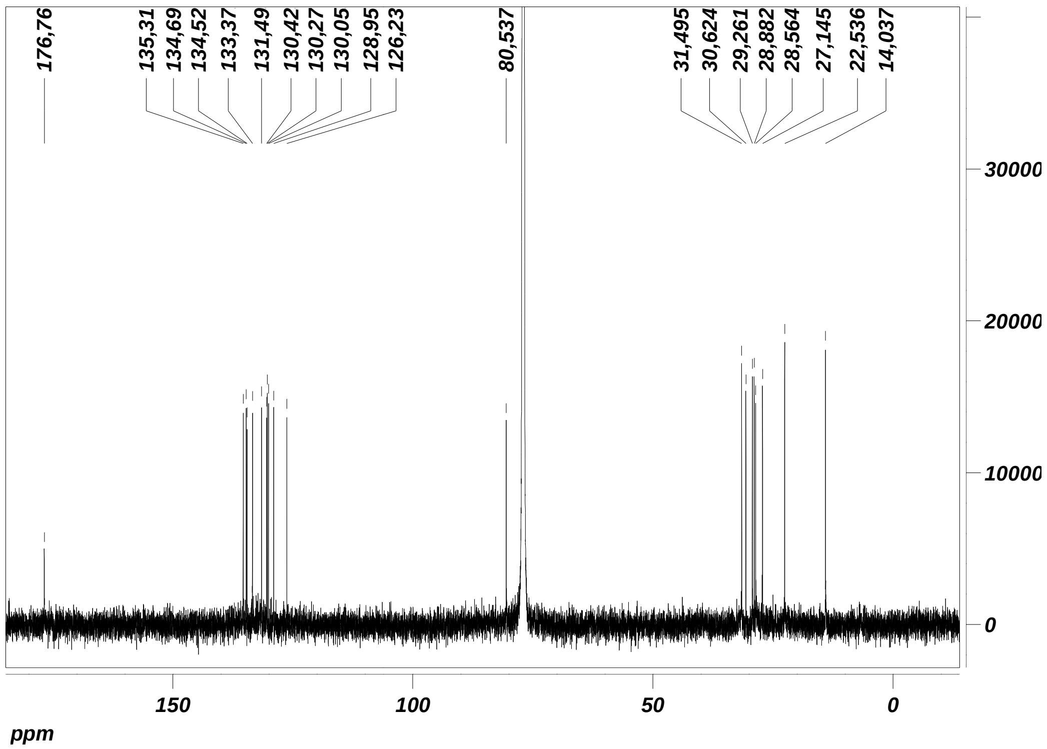

Figure S9: ^{13}C NMR spectrum of compound **6**.

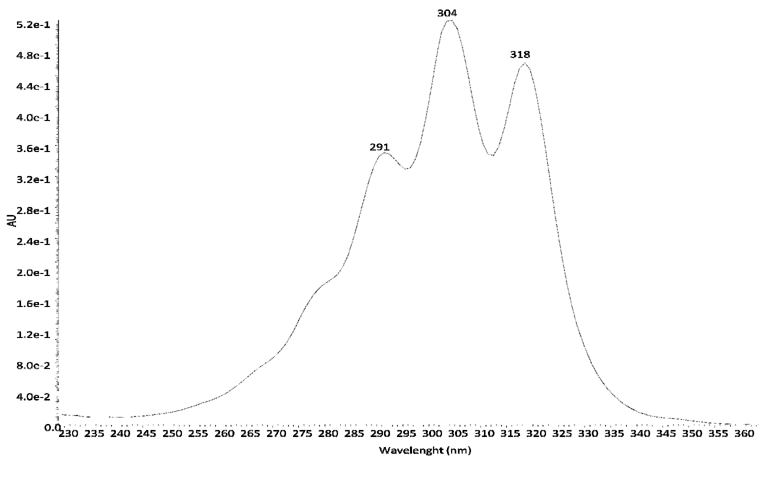

Figure S10: UV–vis spectrum of compound **6** in acetonitrile.
